# Microbiota metabolites in bone: Shaping health and Confronting disease

**DOI:** 10.1016/j.heliyon.2024.e28435

**Published:** 2024-03-20

**Authors:** Dong Han, Weijiao Wang, Jinpeng Gong, Yupeng Ma, Yu Li

**Affiliations:** a Department of Trauma Orthopedics, Yantaishan Hospital, Yantai 264000, China; b Department of Otolaryngology, Yantaishan Hospital, Yantai 264000, China

**Keywords:** Microbiota, SCFAs, Bile acids bone, Tryptophane-derived metabolites, TMAO, Osteoblast, Osteoclast, Osteoporosis, Osteomyelitis, Postbiotics

## Abstract

The intricate interplay between the gut microbiota and bone health has become increasingly recognized as a fundamental determinant of skeletal well-being. Microbiota-derived metabolites play a crucial role in dynamic interaction, specifically in bone homeostasis. In this sense, short-chain fatty acids (SCFAs), including acetate, propionate, and butyrate, indirectly promote bone formation by regulating insulin-like growth factor-1 (IGF-1). Trimethylamine N-oxide (TMAO) has been found to increase the expression of osteoblast genes, such as Runt-related transcription factor 2 (*RUNX2)* and bone morphogenetic protein-2 (*BMP2*), thus enhancing osteogenic differentiation and bone quality through BMP/SMADs and Wnt signaling pathways. Remarkably, in the context of bone infections, the role of microbiota metabolites in immune modulation and host defense mechanisms potentially affects susceptibility to infections such as osteomyelitis. Furthermore, ongoing research elucidates the precise mechanisms through which microbiota-derived metabolites influence bone cells, such as osteoblasts and osteoclasts. Understanding the multifaceted influence of microbiota metabolites on bone, from regulating homeostasis to modulating susceptibility to infections, has the potential to revolutionize our approach to bone health and disease management. This review offers a comprehensive exploration of this evolving field, providing a holistic perspective on the impact of microbiota metabolites on bone health and diseases.

## 1 Introduction

The gut microbiota plays a crucial role in extracting energy from ingested food for the host, facilitating the proliferation of epithelial cells, and preventing the colonization of harmful pathogens [1]. In this sense, metabolites produced by gut microbiota play a critical role in developing the immune system and cellular defense mechanisms [2]. Microbiota metabolites refer to the diverse array of small molecules produced by the complex microbial communities residing in various habitats within the human body, primarily in the gastrointestinal tract [3]. These metabolites play critical roles in maintaining host health and influencing various physiological processes [3]. These metabolites can elucidate the anatomically distant biological effects of gut microbiota, which can impact bone homeostasis through a complex molecular signaling network. This network comprises various components, such as short-chain fatty acids (SCFAs), trimethylamine N-oxide (TMAO), secondary bile acids, and tryptophane-derived metabolites, as documented in previous studies [4,5]. SCFAs are known to control insulin-like growth factor-1 (IGF-1) by inhibiting histone deacetylase (HDAC) and activating receptor-γ as a ligand for G-protein-coupled receptors (GPCRs). Additionally, SCFAs operate as peroxisome proliferators, indirectly promoting bone formation by serving as signal molecules [6,7]. Butyrate can inhibit HDAC in osteoclasts and directly stimulates metabolic reprogramming of osteoclast progenitors. This reprogramming promotes glycolysis at the expense of oxidative phosphorylation and leads to the downregulation of key osteoclast genes, such as tumor necrosis factor receptor (TNFR)-associated factor 6 (TRAF6) and Nuclear Factor of Activated T Cells 1 (NFATC1). Consequently, SCFAs, such as butyrate, play a role in promoting bone homeostasis through a signaling pathway dependent on the gut microbiota [8]. In addition, SCFAs have been found to facilitate the process of bone formation through their ability to decrease the pH levels in the digestive tract [8]. This reduction in pH contributes to a decrease in the formation of calcium and phosphorus complexes, hence promoting the availability of free calcium ions that can be readily absorbed into the bloodstream [8]. Butyrate has been found to increase the surface area available for absorption in the intestine, hence enhancing the absorption of calcium [9]. The process of bone development, which is dependent on parathyroid hormone (PTH), necessitates the generation of butyrate by the gut flora [9]. Furthermore, it has been observed that TMAO enhances the activation of osteoblast genes, such as Runt-related transcription factor 2 (RUNX2) and bone morphogenic protein-2 (BMP-2) [10]. These genes primarily influence bone formation through the BMP/SMADs and Wnt signaling pathways. This finding implies that TMAO facilitates the process of osteogenic differentiation and enhances the overall quality of bone [11].

Microbial-derived metabolites of tryptophan, specifically kynurenine, exhibit a significant association with bone metabolism [12]. Compounds generated through the kynurenine pathway, such as kynurenic acid (KYNA), 3-hydroxykynurenine (3-HKYN), and anthranilic acid (AA), play a significant role in the facilitation of bone-aging features [12]. The impact of kynurenine on the differentiation of bone marrow mesenchymal stem cells into osteoblastic cell lineage has been observed [13]. A high level of peripheral kynurenine has been found to result in the degradation of bone structure by activating the aryl hydrocarbon receptor pathway [14,15]. Additionally, the prooxidative properties of 3-HKYN have been observed to decrease the viability of osteoblast-like cells [16]. Understanding the complex interplay between metabolites produced by microbiota and bone, including their impact on hemostasis and ability to alter susceptibility to infections, can revolutionize current strategies for promoting bone health and managing skeletal system diseases. This review aims to comprehensively examine microbial metabolites' complex and diverse impact on several facets of bone health and disease.

## 2 Gut microbiota and bone

The relationship between gut microbiota and bone health has garnered increasing attention recently. While research has acknowledged the potential influence of microbial communities on bone density and conditions like osteoporosis, the discussion often lacks specificity and depth in exploring these connections [17]. Emerging evidence suggests that certain microbial communities residing in the gut can impact bone density. For instance, specific strains of bacteria, such as *Lactobacillus* and *Bifidobacterium*, have been associated with increased bone mineral density [18]. Conversely, dysbiosis, an imbalance in gut microbiota composition, may contribute to bone loss [18]. Osteomicrobiology focuses on investigating the role of bacteria in bone health, as well as mechanisms by which microbiota influences skeletal development, age-related changes in bone, and pathological bone deterioration [19]. The symbiotic association between the human gut microbiota and the host benefits the dynamic equilibrium that plays a role in bone mass determination [20]. Maintaining a well-balanced and robust microbiome is believed to be crucial in preventing bone loss caused by sex hormone deprivation. This idea is backed by empirical evidence demonstrating that the administration of probiotics to mice that have undergone ovariectomy resulted in the reversal of the pathological progression of osteoporosis [21,22]. Moreover, a comprehensive analysis of multiple studies investigating the quantity and variety of bacterial communities in the gastrointestinal tract of individuals with osteoporosis has revealed a noticeable decrease in microbial diversity among osteoporotic adults [[23], [24], [25]].

The mechanisms underlying the effects of gut microbiota on bone health are complex and multifactorial. One key mechanism involves nutrient absorption, particularly calcium and vitamin D [26]. Gut bacteria play a crucial role in synthesizing and metabolizing these nutrients, which are essential for maintaining bone density. Additionally, gut microbiota can influence hormonal regulation, including the production of hormones such as estrogen and serotonin, which play pivotal roles in bone homeostasis [27]. As an endocrine organ of the body, the gut microbiota may interact with the endocrine system and potentially impact bone homeostasis. Insufficient levels of these hormones induce bone loss and interfere with bone growth [28]. Intestinal permeability and osteoclastic bone resorption are brought on by sex hormone deprivation and are dependent on Tumor necrosis factor (TNF) and Receptor activator of NF-κB (RANK) ligand (RANKL). According to research by Li et al. [29], probiotic *Lactobacillus rhamnosus* GG (LGG) supplementation restores intestinal permeability in the OVX mice model, preventing bone loss caused by sex steroids. Studies have demonstrated that the administration of *Lactobacillus acidophilus* to mice with ovarian cancer or estrogen insufficiency resulted in a decrease in indicators of bone resorption and an improvement in bone growth [30]. Furthermore, probiotic *L. reuteri* treatment stops bone loss in mice with type 1 diabetes and estrogen insufficiency [31,32]. Yan et al. [33] also found that intestinal microbial colonization in germ-free (GF) mice markedly increased the amount of serum IGF-1 and encouraged the development and growth of new bone. Therefore, more research on humans is required to validate the idea that the gut microbiota influences bone metabolism by affecting the actions of several hormones. According to reports, gut microbiota is crucial for the brain system's ability to manufacture hormones and neurotransmitters, including serotonin (5-hydroxytryptamine (5-HT)) [34]. Thus, 5-HT signaling plays a crucial role in controlling the growth and development of bones. According to Ducy et al. [35], the circulation's production of 5-HT may be detrimental to bone metabolism. In contrast, it stimulates the growth of bones when the brain produces it as a neurotransmitter. According to recent research, the gut microbiota plays a part in controlling the levels of 5-HT in the blood [35]. It has been demonstrated that in conditions of animal culture, bacteria like *Streptococcus*, *Corynebacterium*, and *Escherichia coli* can create 5-HT [36]. Furthermore, it has been shown by Sjogren et al. [37] GF mice exhibited elevated trabecular bone/tissue volume and decreased 5-HT levels. A different study showed that intestinal microbiota transplantation can raise the levels of 5-HT in the colon and serum, despite the fact that 5-HT levels are actually decreased in GF animals [38]. According to research by Yadav et al. [39], some spore-forming microorganisms can control gut serotonin, which in turn controls the proliferation of osteoblasts and the creation of bone through the 5-HT Receptor 1B (Htr1b)/PKA/cAMP-response element binding protein (CREB)/cyclins signaling pathway. While discussions often focus on the immune system's role in bone health, it is essential to recognize the microbiota's influence on various hormones involved in bone metabolism [40]. For example, gut bacteria can affect the synthesis of vitamin K, which is necessary for activating osteocalcin, a hormone involved in bone formation [41].

Several lines of research have offered substantial evidence on the role of gut bacteria in bone health management [42]. One mechanism that has been identified is the role of gut bacteria in promoting nutrient absorption and maintaining the integrity of the intestinal barrier, which in turn contributes to the enhancement of bone mineral density (BMD) [42]. Furthermore, microbiota plays a crucial role in modulating the immune system, which in turn plays a pivotal role in maintaining skeletal homeostasis. Tu et al. [43] showed an additional innovative mechanism known as the enteroendocrine-osseous axis, which serves as a comprehensive framework for understanding the interplay between gut microbiota and the endocrine system in facilitating optimal bone health. The gut microbiota influences various hormones involved in bone metabolic control, hence contributing to maintaining skeletal homeostasis [43]. Estrogen's significance in maintaining bone health is widely recognized [44]. Estrogen plays a role in mitigating bone resorption by regulating the balance of T lymphocytes in both systemic circulation and bone marrow [43]. Of note, estrogen directly influences the development and activity of osteoblasts and osteoclasts [45]. The gut microbiota regulates estrogen metabolism, increasing circulating levels. Testosterone, an additional hormone, exhibits the capacity to mitigate the process of apoptosis in osteoblasts while concurrently augmenting the proliferation of osteoblast precursors [43]. Microbiota also affects other vital hormones involved in bone homeostasis, such as IGF-1, parathyroid hormone, serotonin, and gastrointestinal hormones [43]. It is evident that the gut microbiota plays a pivotal role in regulating bone metabolism through its interactions with several physiological systems. In summary, the complex and dynamic correlation between gut microbiota and bone health has emerged as a compelling field of study, providing insights into the extensive influence of the microbiome on skeletal wellness.

As alternatives to the GF mouse model, protocols incorporating broad-spectrum antibiotic (ABX) cocktail formulations have been devised. These procedures are employed to identify the impact of native genetic material on bone metabolism and to reduce or interfere with it [46,47]. In early life (4-week-old mice), subtherapeutic quantities of ABXs can change the composition of the GM, increase BMD, and quicken the formation of new bones [48,49]. Similarly, vancomycin or broadspectrum ABX can improve bone mass when used to eradicate the local microbiota in adult mice that are two months old [33]. The administration of ABX (vancomycin, imipenem/cilastatin, and neomycin) has been shown in a recent study to disrupt gut microbiota immunomodulatory activity and modify postpubertal skeletal development. This is reflected in decreased BMD and increased osteoclastogenesis, but not in osteoblastogenesis or endochondral bone formation [50]. Moreover, disruption of the gut microbiota by ABX can impair osteoimmune interaction by upregulating myeloid-derived suppressor cells (MDSCs) and inhibiting the bone marrow's processing and presentation of major histocompatibility complex (MHC) class II antigen [50]. After ABX treatment, natural gut microbiota recolonization does not appear to be able to rescue bone loss caused by pathological changes in gut microbiota composition (decreased *Bacteroidetes* and increased *Firmicutes*) and disruption of the gut barrier [51].

Despite significant progress, many unanswered questions remain regarding the complex interplay between gut microbiota and bone health. Future research endeavors could focus on elucidating specific bacterial species or metabolites that significantly influence bone density. Additionally, investigating how lifestyle factors like diet and exercise interact with gut microbiota to impact bone health could provide valuable insights. Furthermore, understanding the role of gut-brain communication in regulating bone metabolism represents an exciting avenue for future exploration. We can advance our understanding of the gut-bone axis by addressing these research gaps and developing novel therapeutic strategies for preventing and treating bone-related disorders. In conclusion, while the discussion on gut microbiota's influence on bone health has gained momentum, there remains a need for more specific details and deeper exploration of the underlying mechanisms. By expanding our knowledge in these areas and identifying potential areas for future research, we can further elucidate the intricate relationship between gut microbiota and bone health, ultimately leading to improved therapeutic interventions and better outcomes for individuals at risk of bone-related disorders.

## 3 Physiological and pathological roles of microbiota metabolites in bone health and disease

The intricate and dynamic interplay between microbiota-derived metabolites and bone health has unveiled a fascinating realm of research, elucidating both their physiological and pathological roles in the intricate world of skeletal well-being. These metabolites, which are byproducts of the human microbiota's metabolic activity, play an essential role in molding bone health, contributing to homeostasis maintenance and bone-related disorders' development. This chapter will overview various aspects of microbial metabolites on bone (Table 1).

**Table 1:** Role and action mechanism of microbiota metabolites in bone health and disease.

Microbial metabolites	Type of Study	Method employed	Role	Mechanism	Conclusion	Reference
Propionate and butyrate	*In vivo*	Gas chromatography/mass spectrometry (GC/MS)	Homeostasis	Glycolysis	Osteoclasts' metabolism was reprogrammed by propionate and butyrate, leading to increased glycolysis at the expense of oxidative phosphorylation and the downregulation of important osteoclast genes such as Tumor necrosis factor receptor (TNFR)-associated factor 6 (TRAF6) and Nuclear Factor Of Activated T Cells 1 (NFATc1).	[123]
Propionate and butyrate	*In vitro* and *in vivo*	–	Osteoclast differentiation	GPR41 and GPR109	The study demonstrated the expression of short-chain fatty acid (SCFA) receptors, GPR41 and GPR109, on osteoclast precursors, indicating a potential role for SCFA in regulating osteoclast differentiation.	[233]
Valerate	*In vitro*	–	Maturation of osteoblasts	Nuclear factor kappa B (NF-κB) p65	The pro-inflammatory NF-κB p65 protein synthesis was reduced, and the pro-inflammatory IL-10 expression was increased by valerate. This resulted in the maturation of osteoblasts and the suppression of osteoclast-like cells.	[234]
Acetate, butyrate, and propionate	*In vivo*	High-performance liquid chromatography (HPLC)	Bone formation and growth	Insulin-like growth factor 1 (IGF-1)	Adult bone remodeling was impacted by SCFAs, which had an impact on both bone resorption and creation.	[33]
Butyrate	*In vivo*	LC-MS/MS	Bone formation	Wnt signaling	Butyrate controlled bone anabolism by controlling CD8^+^ T cell Wnt10b production through Treg cell mediation.	[235]
Postbiotics	*In vivo*	–	Bone mineral density (BMD)	–	Postbiotics reduce the amount of bone lost as a result of low estrogen.	[236]
Levulinic acid, and N-acetylneuraminic acid	Clinical	MS	Osteoporosis	–	In postmenopausal women, there were notable alterations in fecal metabolites, gut flora, and fungi, and these alterations were significantly connected with the BMD and clinical findings of the patients.	[237]
Serotonin	*In vitro*	–	proliferation and mineralization	–	Increased serotonin was shown to suppress the growth and mineralization of cells linked to osteogenesis and to positively correlate with modifications in the makeup of faecal metabolites and microbiota.	[238]
Polyamine	*In vivo*	MS/MS	Osteomyelitis	–	It was found that the role of gut microbiota metabolites in regulating bacterial infections that spread beyond the gut and using polyamines as a supplemental treatment for osteomyelitis in obese and type 2 diabetic (T2DM) patients.	[239]
Bile acid	Clinical, and *in vitro*	–	BMD	beta-C-terminal telopeptide (b-CTX)	In postmenopausal women, serum bile acid showed a negative correlation with bone turnover indicators that suggest bone absorption and a positive correlation with BMD.	[109]
Ursodeoxycholic acid (UDCA)	*In vivo*	Nuclear magnetic resonance (NMR) spectrometer	Bone regeneration	Hydrogen peroxide (H2O2)	PUDCA showed a considerably greater ability for bone regeneration and anti-inflammatory effects than equal doses of UDCA in rat models of bone deficiency.	[218]
Bile acids	*In vitro*	LC-MS and GC-MS	Rheumatoid arthritis (RA)	Bone erosion	Disrupted microbial bile acid metabolism mediates the relationship between the antigenic response and bone disintegration in RA and the gut microbiome.	[176]
Glycoursodeoxycholic acid (GUDCA)	Clinical and *in vitro*	HPLC	Osteoporosis	Osteoblast and osteoclast differentiation	the effect of conjugated and unconjugated bile acids on osteoblast and osteoclast development that were detected in the serum of PSCOPO patients.	[240]
Bile acid	Clinical and *in vitro*	–	T2DM	Bone mineral density	This study illustrated the possible contribution of bile acids on T2DM patients' bone metabolism.	[238]
Serotonin	Clinical and *in vitro*				Increased serotonin was shown to suppress the growth and mineralization of cells linked to osteogenesis and to positively correlate with modifications in the makeup of faecal metabolites and microbiota.	
Lipopolysaccharide (LPS) and Peptidoglycan (PGN)	*In vivo*	–	Bone resorption and osteoclastogenesis	Toll-like receptor (TLR) 2 and TLR4	Inducing bone resorption and osteoclastogenesis, either gram-positive or gram-negative PGN collaborated with LPS, potentially through coordinating the actions of TLR2, NOD1, NOD2, and TLR4 signaling.	[241]
LPS	*In vitro*	–	Osteoclast formation	Tumour Necrosis Factor alpha (TNF-a)	It was strongly suggested that LPS induced the production of osteoclasts in RAW 264.7 cells and promoted the formation of Tartrate-resistant acid phosphatase (TRAP)-positive multinucleated giant cells (MGC) in these cells.	[194]
LPS	*In vitro*	–	Osteoclastogenesis	TLR4	Macrophages primed with Receptor activator of NF-κB (RANK) ligand (RANKL) and treated with LPS exhibit regulated osteoclastogenesis due to the secretion of TNF-α via LPS/TLR4 signaling.	[242]
LPS	*In vitro*	–	Osteoclast differentiation and activation	Mitogen-activated protein kinase (MAPK) and Cyclooxygenase-2 (COX-2)	LPS increased COX-2 expression and RANK signaling, encouraging osteoclast activation and differentiation.	[243]
LPS	*In vitro*	–	Bone formation	BMP-2 and TGF-b1	By generating IL-1b, LPS inhibited the ectopic bone growth caused by BMP-2 and transforming growth factor beta-1 (TGF-b1).	[244]
LPS	*In vivo*	–	BMD	–	In rodent models, exogenous LPS caused changes in bone structure and BMD; however, a well-defined model of exogenous LPS-induced bone loss is still pending.	[245]
LPS	*In vitro*	–	Osteogenic differentiation	TLR4	LPS reduced the osteogenic capacity of human PDLSCs by activating the TLR4-regulated NF-κB pathway.	[246]
LPS	*In vivo*	–	Infection		Gram-negative periprosthetic joint infections (PJI) signal a greater chance of aseptic loosening upon reimplantation, primarily because of LPS-mediated effects on osteoclast development.	[247]

### 3.1 The relation between microbiota metabolites and mineral absorption

The latest studies have shed light on the crucial significance of microbial metabolites in modulating the process of mineral absorption in the gastrointestinal tract, thus impacting the maintenance of bone health and homeostasis [41]. The gut microbiota can influence the absorption of micronutrients and the synthesis of vitamins crucial for maintaining optimal bone health. Calcium is a fundamental nutrient that plays a pivotal role in maintaining bone homeostasis [41,52]. Approximately 99% of the total calcium content in the human body is mainly concentrated inside the skeletal system [52]. Calcium shortage is associated with significant bone demineralization and catalyzes the onset of osteoporosis [53]. The gut microbiota has been found to facilitate the absorption of calcium by producing certain metabolites, such as SCFAs [54]. The lowering of pH in the intestinal lumen is one of the impacts of a high concentration of SCFAs in the gastrointestinal tract. This decrease in pH has been seen to increase mineral solubility while inhibiting the formation of calcium complexes, notably calcium phosphate [54]. Therefore, SCFAs have been shown to enhance the availability of calcium and promote its absorption. In contrast, it has been observed that SCFAs also have the ability to improve the paracellular transport of calcium across the intestinal epithelium [55]. The gut microbiota promotes bone mineralization and subsequent bone growth by enhancing calcium absorption [54,55]. A favorable correlation was observed between the quantity of *Bifidobacterium* and BMD [56]. *Bifidobacterium* is a symbiotic bacterium residing in the gut microbiota and serves a crucial function in enhancing intestinal health and fortifying gut barrier activities by producing SCFAs [57]. Changes in *Bifidobacterium* levels can potentially reduce SCFA synthesis, disturb the integrity of the intestinal barrier, limit calcium absorption, and eventually contribute to bone loss among individuals with osteoporosis.

The gut microbiota can influence the synthesis of a crucial vitamin necessary for maintaining optimal bone health, namely vitamin K [58]. Vitamin K has been found to enhance bone production by facilitating osteoblast differentiation and inhibiting osteoclast differentiation [52,59,60]. In this scenario, vitamin K is a cofactor in synthesizing crucial proteins that are particular to bone tissue, such as osteocalcin and gamma-carboxyglutamate protein. Research findings have shown that around 50% of the vitamin K that is ingested is produced through the synthesis activities of our gut microbiota, including *Bacteroides* [58]. The colon, a significant provider of nutrients including vitamins B and K, can influence the development and maintenance of bone mineral density, either directly or indirectly [61]. The bone matrix contains a substantial quantity of osteocalcin, a non-collagenous protein. Supplementary with vitamin K, this protein is alternatively referred to as bone Gla-protein [62,63]. It is a prerequisite for this procedure to be initiated. This component's carboxylation is crucial for binding this protein to bone minerals. While vitamin K can be acquired through dietary sources, it is vital to note that intestinal production significantly contributes to the vitamin supply. If the gut microbiota cannot regulate the number of beneficial microorganisms, an excess of uncarboxylated osteocalcin in circulation may result from a decrease in vitamin K production. The absence of carboxylated osteocalcin in the bone matrix can result in bone tissue deterioration and increased susceptibility to fracture [63,64]. In brief, the impact of microbial metabolites on the assimilation of vital minerals, specifically calcium and phosphorus, constitutes a crucial determinant in preserving skeletal equilibrium and general skeletal well-being. Researchers aim to uncover novel strategies for improving bone regeneration, alleviating bone diseases, and promoting general musculoskeletal health by clarifying how these metabolites affect mineral absorption [65]. The inherently dynamic character of this field of study has the potential to radically revolutionize our approach to bone health and the management of bone-related diseases.

### 3.2 The relation between microbiota metabolites and osteogenic differentiation, chondrogenesis, bone formation, and resorption

Recent research has indicated that the presence of microbial metabolites at physiological levels can potentially improve the characteristics and behavior of osteoblasts, which are responsible for bone formation [66]. Multiple studies have demonstrated that the presence of butyrate, within the concentration range of 500 nM to 1 mM, leads to an elevation in alkaline phosphatase (ALP) production in murine calvarial organ cultures [67]. It has been observed that butyrate promotes the transcription of Runx2 in MC3T3-E1 cells [67]. Furthermore, butyrate has been found to enhance the synthesis of osteoprotegerin (OPG). The osteoclastogenesis inhibitory decoy receptor, known as OPG, functions as a binding site for RANKL, a pivotal component in osteoclast development. OPG indirectly increases bone formation by decreasing osteoclast activity via its interaction with RANKL [68]. Previous research has indicated that sodium butyrate exhibits osteogenic properties in human amniotic membrane-derived mesenchymal stem cells (MSCs) [69]. When exposed to elevated concentrations of SCFAs that surpass physiological levels, osteoblasts exhibit distinct changes in their phenotypic and survival. The alterations include cytotoxicity, which pertains to the deleterious impacts on cellular viability, and an augmentation in the synthesis of RANKL [66]. Valproate, a well-known inhibitor of HDAC, exhibited a noteworthy effect on promoting osteogenic differentiation. The observed impact was shown to be contingent upon the dosage of valproate, as it resulted in the increased expression of osteogenic genes such as osterix, osteopontin, BMP-2, and Runx2 [70].

The process of chondrogenic differentiation of MSCs originating from bone marrow (BM-MSCs) and the subsequent development of callus play a vital role in initiating effective healing of fractures through the endochondral pathway [66]. A study conducted by Garrison et al. [71] examined the influence of sodium butyrate on the differentiation of embryonic limb bud cells. Their study revealed that butyrate concentrations within the 0.03–1 mM range inhibited chondrogenesis in micromass cultures. Paradis et al. [72] demonstrated that valproate can decrease the expression of *Sox9* and *Runx2*, which are crucial regulators of chondrogenesis and osteogenesis in mice. Aulthouse et al. [73] found that valproate also leads to a reduction in the production of chondrogenic markers, specifically type II collagen and sulfated proteoglycan, in human chondrocytes. Pirozzi et al. [74] demonstrated that butyrate inhibits highly influential inflammatory signaling pathways by decreasing the production of pro-inflammatory cytokines in murine chondrocytes. This inhibition occurs through a pathway mediated by GPR43. Furthermore, it has been demonstrated that butyrate can restrict the synthesis of catabolic matrix metalloproteinases (MMPs) and mitigate the breakdown of type II collagen generated by inflammation in explant culture [75]. Anti-inflammatory effects of butyrate in human chondrocytes were discovered to be unrelated to the NF-κB DNA binding activity, as demonstrated by Chabane et al. [76]. This finding is significant considering the crucial role of NF-κB in mediating the production of the highly potent pro-inflammatory cytokine IL-1β in chondrocytes.

The relationship between bone production and resorption is a dynamic and highly regulated mechanism, which is crucial for preserving skeletal integrity. Recent studies have revealed the captivating function of microbial metabolites in exerting influence over these crucial systems. Notably, research has demonstrated that butyrate has a role in enhancing the differentiation of stromal cells towards osteogenic lineage and facilitating the creation of mineralized nodules [68,77]. Furthermore, the augmentation of SCFA production by using oligosaccharide dietary supplements was associated with an increase in BMD [78]. In contrast, it has been observed that supplementation of SCFAs reduces bone volume in mice treated with antibiotics while not affecting bone turnover rates [79]. The findings mentioned above have generated a necessity to investigate the impact of SCFAs on bone volume in mice possessing typical gut microbiota. Experimental evidence supports the idea that butyrate's capacity to trigger bone development is due to an increase in the number of regulatory T cells (Tregs) in the bone marrow. Indeed, research involving the administration of anti-CD25 antibodies to impede the proliferation of Tregs has demonstrated that the ability of butyrate to stimulate bone formation and enhance bone density is compromised in the absence of Tregs [80]. This was confirmed by utilizing DEREG mice, a genetically modified strain expressing the human diphtheria receptor, specifically in Tregs. The administration of diphtheria toxin to DEREG mice results in the elimination of Tregs.

Similarly, it has been observed that the administration of butyrate cannot stimulate bone production or enhance bone density in DEREG mice that have been subjected to diphtheria toxin treatment. The absence of any obvious symptoms of increased inflammation in these experiments conclusively ruled out the possibility that Treg cell reduction hampered butyrate's bone-strengthening benefits by inducing an inflammatory response. The administration of a partial blockage of Tregs effectively inhibited the upregulation of Wnt10b expression in CD8^+^ T cells caused by butyrate. The expression of Wnt10b has been observed to enhance the proliferation, differentiation, and survival of osteoblasts while also playing a role in the regulation of osteoprotegerin synthesis [[81], [82], [83], [84]]. In human biology, Wnt10b has been identified as a reliable indicator of bone mass [85]. Conversely, in mice, Wnt10b plays a crucial role in developing bone mass under normal circumstances, and its absence leads to the progressive loss of bone density as individuals age [[86], [87], [88]]. The involvement of Wnt10b as an intrinsic Wnt ligand in bone function is supported by the fact that heterozygous *Wnt10b* ± mice have significantly less trabecular bone [88]. Furthermore, it has been found that a specific subset of immune cells known as CD8^+^ T cells produce a pool of Wnt10b that is essential for inducing bone formation in response to PTH.

Several studies have demonstrated that SCFAs can impede the process of osteoclastogenesis [66]. Adding 0.5 mM sodium butyrate to rat bone marrow cultures resulted in a significant decrease in osteoclast development. Specifically, there was a 98% reduction in the number of Tartrate-resistant acid phosphatase (TRAP)-positive multinucleated cells resembling osteoclasts. The notable impacts of sodium butyrate on osteoblastic and osteoclastic cells were previously documented. Specifically, it was observed that a concentration of 0.5 mM sodium butyrate led to an increase in ALP activity in developing osteoblasts. Nonetheless, the precise timing of sodium butyrate supplementation was revealed as a critical component since ALP activity only increased when delivered to the cells before the confluence. In addition to its role in promoting osteoblast development, the administration of sodium butyrate to bone marrow cells decreased the production of TRAP-positive multinuclear cells. The observed reduction can be ascribed to the cytotoxic effect of sodium butyrate within the concentration range of 0.25 mM–2.5 mM [89].

The differentiation and activation of osteoclasts are contingent upon the signaling pathway mediated by RANK upon stimulation with its ligand, RANKL [90]. Osteopetrosis is observed in mice with a deletion of the *NF-kB* gene, which is a crucial signaling protein associated with RANKL. Rahman et al. [90] indicated that the transcriptional activity dependent on NF-kB is progressively suppressed dose-dependent upon treatment with these drugs. The nuclear protein level of NF-kB exhibited a reduction after activation. The involvement of NF-kB in the differentiation and maturation of osteoclasts has been widely recognized. Additionally, it has been observed that HDAC inhibitors effectively suppress both preosteoclast production and fusion, which are crucial events in osteoclast differentiation [90]. The observed inhibitory effect of these drugs on osteoclastogenesis appears to be mediated through their inhibitory effects on NF-kB activation. This study introduces the new impact of two HDAC inhibitors, trichostatin A (TSA) and sodium butyrate, on the process of osteoclastogenesis. Osteoclasts play a critical role in the physiological process of bone remodeling and also contribute to the pathological bone loss observed in inflammatory conditions such as rheumatoid arthritis and periodontal disease. In addition, they play a role in postmenopausal osteoporosis. Rahman et al. [90] provided a new potential approach for identifying targeted therapeutic agents for the treatment of these diseases.

Osteoclasts are a type of cells with many nuclei that play a crucial role in both physiological and pathological processes of bone resorption [4]. SCFAs have been detected to attenuate the process of osteoclast development [89]. One of the mechanisms through which this effect is achieved is the inhibition of HDAC activity [91,92]. Butyrate and TSA have been shown to prevent osteoclast development in primary bone marrow cells [93]. The administration of the more recent HDAC inhibitor, depsipeptide FR901228, provided more evidence supporting the antiosteoclastic effects of butyrate. This finding implies a potential novel application of HDAC inhibitors as medicines for reducing bone resorption [94]. Two further studies found that butyrate and, to a lesser extent, propionate had antiosteoclastic effects, preventing the growth of osteoclasts [95,96]. The inhibition of osteoclast differentiation is particularly effective when SCFAs or HDAC inhibitors are introduced during the first stages of osteoclast differentiation [93,97,98].

Bone loss was prevented in FFR1 (GPR40) knockout mice because osteoclast production was stymied. FFR1 (GPR40) is a receptor that binds mid-to long-chain fatty acids [99]. A study conducted by Lucas et al. [98] served as a catalyst for further explorations into the possible impacts of high-fiber meals (prebiotic), bacterial transfer (probiotic), or SCFA supplementation (postbiotic) on bone metabolism in both normal and osteoporotic conditions [98]. Lucas et al. [98] also demonstrated a decrease in osteoclast populations in both C57BL/6 mice and osteoporotic mice after being treated with propionate and butyrate. In summary, microbial metabolites have diverse effects on bone health, impacting the processes of osteogenic differentiation, chondrogenesis, bone production, and resorption. Modifying these metabolites exhibits potential for prospective therapeutic interventions to augment musculoskeletal well-being, mitigate bone disorders, and redefine our approach to skeletal health.

### 3.3 Microbiota metabolites and bone metabolism

In recent years, there has been a noticeable rise in the emphasis placed on the research of bone metabolism and its intricate interplay with the gut microbiota and its associated metabolites. During the process of intestinal transit, the conjugated primary bile acids undergo uncoupling and dehydrogenation events inside the gut microbiota [100]. This produces secondary bile acids, including deoxycholic acid and lithic bile acids [101,102]. Bile acids can have a significant impact on bone metabolism. The findings of a study carried out on a cohort of postmenopausal women demonstrated a positive link between blood concentrations of bile acids and BMD.

Conversely, a negative association was detected between bile acids and markers of bone resorption [103]. The gut microbiota can modify the quantity and composition of secondary bile acids through signaling through the farnesoid X receptor (FXR) and the G protein-coupled bile acid receptor 5 (TGR5), leading to diverse metabolic consequences [104]. The FXR protein functions as a sensor for bile acids, regulating the balance of bile acid levels in the body, and also plays a significant part in the process of bone metabolism [105]. The findings from *in vitro* experiments indicate that bile acids can modulate bone metabolism by activating FXR signaling and upregulating Runx2 expression. Additionally, bile acids can enhance extracellular signal-regulated kinase (ERK) and β-catenin signaling pathways [105]. Secondary bile acids have been identified as agonists of TGR5, a receptor involved in regulating various physiological processes. Secondary bile acids indirectly affect bone metabolism by promoting the manufacture of GLP-1, a hormone with established effects on bone health, via TGR5 [106].

The maintenance of bone health is significantly impacted by both primary and secondary bile acids [107,108]. Serum bile acid levels in postmenopausal women are inversely correlated with β-CTX concentrations and favorably associated with BMD of the lumbar spine, femoral neck, and whole hip [109]. Following OVX288 treatment, female mice experiencing bone loss had lower levels of DCA. By simultaneously stimulating bone production and suppressing bone resorption, DCA supplements can halt bone loss [110,111]. These findings show DCA's ability to protect bones even more. On the other hand, LCA, a different form of secondary bile acid produced mainly by gut bacteria dehydroxylate proteins, seems to have a negative impact on bone. The biological action of 1,25-hydroxyvitamin D, a bone-strengthening supplement, is mediated by gene transcription that occurs downstream of the vitamin D (VD) receptor (VDR). Potent VDR agonist LCA inhibits VD-mediated stimulatory effects on osteoblast activity by promoting the expression of the CYP24A gene, which codes for the hydroxylase that catabolizes vitamin D. This mechanism explains how LCA diminishes VD-mediated stimulatory effects on osteoblast activity in competition with 1,25-hydroxyvitamin D [112]. Two crucial regulatory elements for bile acid-mediated bone metabolism are the bile acid nuclear receptor FXR and the bile acid membrane receptor TGR5. The AMP-activated protein kinase (AMPK) signaling pathway is responsible for the increased osteoclast development observed in aged and OVX Tgr5 knockout mice and decreased bone mass [113]. FXR is a bile acid sensor associated with bone metabolism. Upregulating BMP-2 expression, activating the ERK and β-catenin signaling pathways, and promoting Runx2 expression can improve osteoblast development and reduce osteoclast differentiation [114,115]. Bile acids are also essential for the gut's absorption of calcium. For instance, whereas LCA has the opposite effect, DCA can decrease Ca2+ absorption [116,117]. At least somewhat, the impact of bile acids on Ca2+ absorption can also affect bone health.

Due to their strong regulatory ability, SCFAs have generated a great deal of attention in the study of modifying bone metabolism [118]. Beneficial bacteria create SCFAs, which can inhibit HDACs, activate GPCRs, and function as energy substrates [119]. By interfering with the TNF-α-induced nuclear translocation of NF-κB and the RANKL-mediated activation of the p38 MAPK signaling pathway, butyrate, an SCFA, can decrease osteoclast development. The primary receptors for SCFAs are GPCRs, such as GPR109, the butyrate and niacin receptor; GPR41, the propionate and butyrate 258 receptors; and GPR43 and Olfr78, the acetate and propionate receptors [[120], [121], [122]]. Lucas et al. showed that the early metabolic reprogramming of preosteoclasts—which changed cellular metabolism from oxidative phosphorylation (OXPHOS) to glycolysis during the early stages (24–48 h) of osteoclast differentiation—was necessary for the direct influence of SCFAs on bone homeostasis [123]. This implies that by modifying cell metabolism *in vivo*, SCFAs may impact bone homeostasis. SCFAs can stimulate Treg differentiation, proliferation, and expansion.

The regulation of several cellular processes, such as osteoclast differentiation, is significantly influenced by cell metabolism [98]. This differentiation process involves a series of progressive metabolic alterations. The maturation of osteoclasts from precursor cells is reliant on oxidative phosphorylation, while the process of bone resorption by mature osteoclasts is dependent on glycolysis. Lucas et al. [98] demonstrated that the stimulation of propionate/butyrate resulted in a considerable increase in glycolysis in osteoclast precursors after 48 h. However, no significant changes were observed in oxidative phosphorylation. Indeed, previous studies have demonstrated a correlation between enhanced glycolysis and the downregulation of TRAF6 in many cell types [124]. Despite the presence of SCFA receptors on osteoclasts, the metabolic changes mentioned above were observed in studies involving osteoclast assays of mice with single deletion (KO) of GPR41, GPR43, and GRP41/43. Some analyses were employed to assess the impact of propionate and butyrate administration on energy stress. Specifically, the activation of AMP-activated protein kinase (AMPK) was investigated, which resulted in the observation of an elevated pAMPK/AMPK ratio. Notably, in the presence of propionate or butyrate, the targeted suppression of glycolysis using 2-deoxy-d-glucose (2DG) primarily during the first 48 h of osteoclastogenesis effectively counteracted the suppressive effects of propionate and butyrate. The introduction of propionate and butyrate at later stages did not have any statistically significant impacts on the process of osteoclast development [98]. These findings proved that the administration of propionate and butyrate to osteoclast precursor cells alters their metabolic processes during the first stages of osteoclast differentiation, leading to a shift toward glycolysis [98]. This metabolic change induces cellular stress, ultimately impeding the process of osteoclast differentiation. This emerging field of research highlights the profound impact of microbial metabolites on the metabolic process of bone, hence opening the door to innovative therapeutic interventions and strategies for enhancing musculoskeletal health and treating bone-related diseases. The study of microbial metabolites in the context of bone metabolism is a new area of study in the field of musculoskeletal medicine, with various potential opportunities for future research and advancement.

### 3.4 Microbial metabolites and insulin-like growth factor

IGF, namely IGF-1 and IGF-2, are endocrine hormones significantly influencing tissue growth and development, particularly concerning skeletal structures [79]. Recent research suggests that the gut microbiome can alter IGF signaling and bone health through various methods. The addition of conventional microbiota to GF flies increased insulin/IGF-like peptide activity, and IGF-1 was shown to mediate the impact of microbiota on postnatal growth [125,126]. IGF-1 is a growth factor affecting bone via endocrine and paracrine/autocrine pathways [127]. Mice that exhibit a deficit in liver-specific IGF-1 exhibit rather normal growth and development despite a significant reduction of 75% in the levels of circulating IGF-1. This discovery suggests that the generation of IGF-1 within the liver has a significant impact on the promotion of bone development [128]. Furthermore, IGF-1 can drive bone production and resorption by directly influencing osteoblasts and osteoclasts [129,130]. Elevating blood IGF-1 levels is one proposed route by which bacteria may accelerate bone development in adult mice. Because a significant proportion of Gram-positive bacteria engage in the production of SCFA through fermentation, this process has been detected to have an impact on the composition of the gut microbiota, leading to decreased levels of IGF-1 and procollagen type 1 N-terminal pro-peptide (P1NP). The focus of the study conducted by Yan et al. [79] was the synthesis of SCFAs as a possible mediator of the observed effects. Research findings demonstrated that the exclusive use of vancomycin, an antibiotic, led to a significant decrease in the concentrations of SCFAs in the cecum. On the contrary, germ-free animals exhibited a propensity for higher SCFA concentrations within one month of colonization. By supplementing antibiotic-treated mice with SCFAs, the effects of colonization on serum and tissue IGF-1 levels, in addition to bone mass, were replicated. Further research employing GF mice that were administered SCFA or animals devoid of SCFA receptors may contribute to a better understanding of the effects of SCFA on bone. Collectively, Yan et al. [79] found that SCFAs produced by gut bacteria play a role in microbiota-induced changes in IGF-1 levels in the host, as well as the impact of colonization on the process of bone remodeling. Yan et al. [52] aimed to elucidate further the potential processes that establish a connection between microbiota and bone health. They specifically examined the direct influence of SCFA on osteoclasts and the possibility of SCFA-induced serotonin increase. Serotonin, a microbiota-influenced circulating neurotransmitter, has signaling capabilities that may contribute to this link. While butyrate and propionate have been shown to inhibit osteoclast formation, the impact of these molecules on osteoclasts *in vivo* is unlikely due to lower circulating concentrations of these molecules compared to the concentration required to affect osteoclast differentiation [52]. Despite increased osteoclast production, mice lacking the GPR109 gene have a slight gain in bone mass. According to this finding, Butyrate and GPR109 may play nonosteoclast roles in bone modulation. The increase in trabecular bone mass reported in mice lacking GPR109 is comparable to that observed in GF animals, which have lower amounts of butyrate and, thus, less GPR109-mediated signaling [79]. Although SCFA may directly impact the functioning of specific bone cell types, it is more likely that an indirect mechanism is at work due to the comparatively low quantities of SCFA in circulation. Given the rapid progress in understanding host-microbiota interactions and metabolomics, it is likely that further possible pathways clarifying the impact of microbiota on bone health will be revealed in the near future. Microbial metabolites, especially SCFAs, significantly impact skeletal integrity when combined with IGF-1. Osteocytes influence several aspects of bone health, including bone density, formation, and remodeling. The complicated connection between the gut microbiota and IGFs in regulating bone metabolism is an enthralling subject of study that holds promise for creating novel ways to improve bone health and treat diseases such as osteoporosis.

### 3.5 Immunomodulatory functions of microbiota metabolites in bone health

**Fig. 1:**
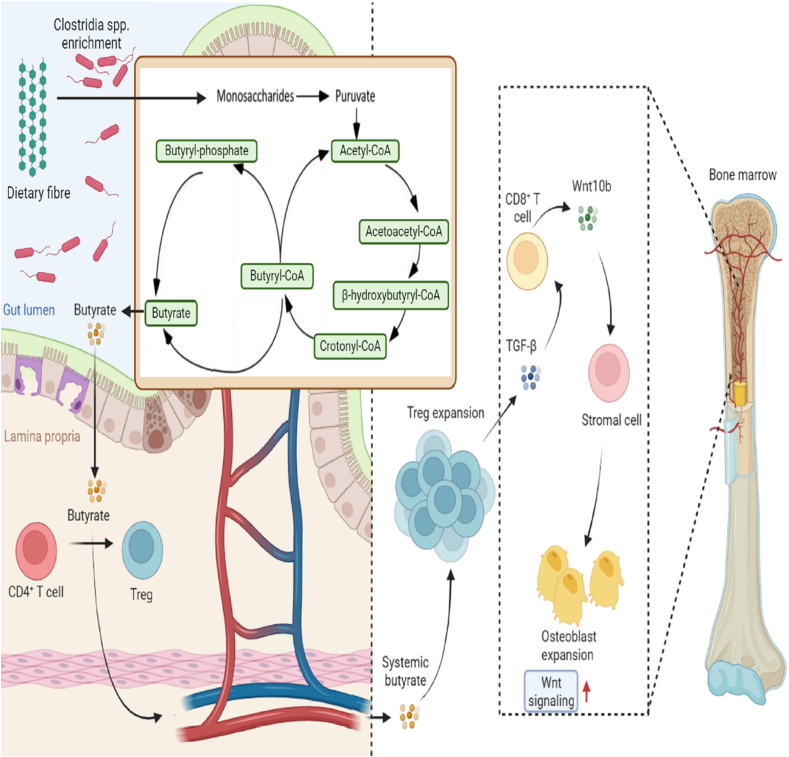
**Microbial Butyrate Increases Bone Mass by Enhancing Wnt Signaling.** Microbial butyrate, whether administered directly to mice or produced by the microbiome, plays a role in promoting the expansion of T regulatory (Treg) cells. These Treg cells, in turn, produce transforming growth factor-beta (TGF-b) within the bone marrow. The TGF-b produced by Treg cells in the bone marrow contributes to the release of Wnt10b by bone marrow-resident CD8^+^ T cells. This cascade of events ultimately leads to enhanced bone anabolism. In summary, the mechanism outlined in the text proposes that microbial butyrate influences the immune response by promoting the expansion of Treg cells. The Treg cells, through the production of TGF-b, impact the bone marrow environment, leading to increased release of Wnt10b by CD8^+^ T cells. The enhancement of Wnt signaling, in turn, is associated with increased bone anabolism, suggesting a potential link between microbial butyrate, immune regulation, and bone mass.

Recently, there has been a rising emphasis on studying the relationship between gut microbiota and various aspects of human health [80]. An area of research that carries substantial importance is the analysis of microbial metabolites' effects on bone health, specifically emphasizing their immunomodulatory characteristics. Tyagi et al. [80] demonstrated that the administration of LGG resulted in an elevation in butyrate concentrations in both the gastrointestinal tract and the bloodstream. Additionally, LGG supplementation led to an increase in the population of Treg cells in the bone marrow. These effects were found to be associated with an enhanced synthesis of the Wnt ligand Wnt10b. Mechanistically, this upregulation of Wnt10b was attributed to the augmented binding of NFAT1 and SMAD3 transcription factors to the Wnt10b promoter. According to a study carried out by Lucas et al. [98], it has been observed that the anti-resorptive properties of propionate or butyrate are not affected by T cells. These data suggest that the ability of butyrate and LGG to promote bone formation is dependent on the presence of Treg cells and CD8^+^ T cells (Fig. 1). According to the findings of Roser-Page et al. [131], activation of T cell receptors (TCRs) is required for Wnt10b expression in CD8^+^ T cells. In the setting of live organisms, CD8 T cell activation is thought to be predominantly driven by CD8^+^ T cells, with modest auto-reactivity activated by homeostatic T cell renewal processes [132]. The activation of CD8 T cells is also influenced by endogenous antigens of a microbial nature naturally absorbed from the gastrointestinal tract. In the context of T cell activation, it has been discovered that NFAT preferentially associates with AP-1 [133,134]. According to a study performed by Macian et al. [135], Treg cells) have been found to inhibit the production of AP-1 and facilitate the association between NFAT and SMADs. The ChIP data presented in their study provides evidence that LGG and butyrate enhance the binding of NFAT1 and SMAD3 to the Wnt10b promoter. However, this effect is observed only in the presence of an elevated number of Treg cells. A study conducted by Macian et al. [135] revealed that NFAT2 demonstrated a notable affinity for binding to the Wnt10b promoter, particularly in activated OT-1 CD8^+^ T cells.

The population of CD14^+^ monocytes/macrophages, which serve as progenitor cells for osteoclasts, exhibited a considerable reduction in the bone marrow after the injection of butyrate compared to mice in the control group and those treated with antibiotics. Previous research has shown that the effect of butyrate on CD45^+^CD14^+^ cells results in a direct decrease in CD14 receptor expression via posttranscriptional pathways [136]. Furthermore, SCFA propionate was found to affect bone marrow hematopoiesis in mice by altering the formation of macrophage and dendritic cell precursors [137]. Previous research has revealed that SCFAs affect macrophages. SCFAs have been reported to improve macrophage phagocytic capacity and antibacterial activity [138,139]. In contrast to the observed decrease in the population of monocytes/macrophages, butyrate administration increased the number of CD19^+^ B cells in the bone marrow when compared to control and antibiotic-treated mice. A recent study demonstrated that butyrate had a suppressive effect on arthritis in mice and that this action depended on regulatory B cells (Bregs) [140]. Breg cells are essential in the process of bone union because they limit the release of proinflammatory cytokines [141]. Previous research has connected reduced Breg cell function to delayed healing in individuals with tibial fractures [142].

Butyrate affected serum inflammatory indices mainly by reducing the proinflammatory interleukin (IL)-6 level. IL-6 is produced not only by immune cells, primarily macrophages, but also by osteoblasts and promotes osteoclast formation [143]. Previous research [144] found that using an intervention targeting soluble IL-6 improved recovery of defective fracture repair in mice following severe trauma. TNF-α, IL-1β, and IL-17 are some of the other cytokines with osteoclastogenic potential [145]. The administration of Rifampin + Levofloxacin to mice significantly reduced the levels of SCFAs, including acetate, propionate, and butyrate, in the cecal water. The observed change in SCFA concentrations was concurrent with a significant variation in the composition of their gastrointestinal microbiota [146]. The potential association between reduced SCFA levels and increased concentrations of proinflammatory cytokines, including TNFα, IL-17a, and IL-17f, in the circulatory system of mice exposed to antibiotic therapy may be elucidated [146]. In summary, compared to mice in the control and antibiotic groups, butyrate administration reduced monocyte/macrophage populations in the bone marrow and decreased systemic levels of IL-6 in a murine osteotomy model. In contrast, the mice that were administered antibiotics had reduced concentrations of SCFAs in the cecum, accompanied by heightened levels of circulating proinflammatory cytokines such as TNF-α, IL-17a, and IL-17f. In brief, identifying microbial metabolites, specifically SCFAs, as influential factors in influencing the immune system is increasing, highlighting their importance in preserving good bone health. Metabolites have been found to significantly impact immune system activity, T-cell functioning, and inflammatory reactions. The impacts mentioned above have significant consequences pertaining to the preservation of bone density, the mechanism of bone formation, and the alteration of bone tissue.

### 3.6 Microbiota metabolites affect bone healing

**Fig. 2:**
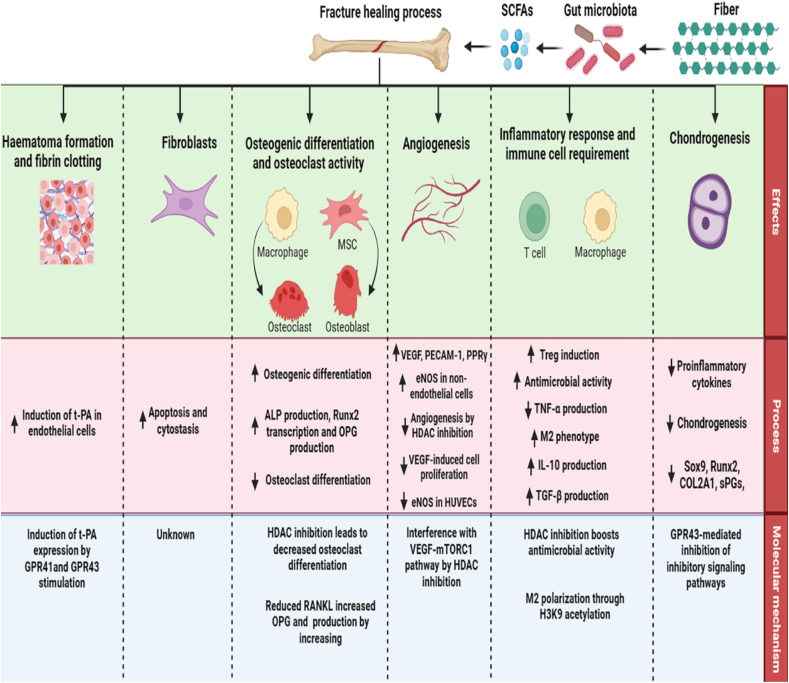
**Role and function of microbiota short-chain fatty acids (SCFAs) on bone fracture healing.** The gut microbiota plays a crucial role in maintaining bone health through the fermentation of dietary fibers to produce SCFAs. Microbiota-derived SCFAs play a crucial role in bone fracture healing by modulating various cellular processes and molecular mechanisms. 1) Hematoma Formation and Fibrin Clotting: SCFAs, acting through G protein-coupled receptors (GPR41 and GPR43), induce tissue plasminogen activator (t-PA) expression in endothelial cells. This induction enhances fibrinolysis, promoting the breakdown of fibrin clots and facilitating hematoma resolution, which is essential for the subsequent stages of bone healing. 2) Fibroblast Function: SCFAs regulate fibroblast behavior by modulating apoptosis and cytostasis. They promote fibroblast proliferation and migration, facilitating the formation of granulation tissue and wound closure during the early stages of fracture healing. 3) Osteogenic Differentiation and Osteoclast Function: SCFAs influence osteogenic differentiation by promoting the expression of alkaline phosphatase (ALP), Runx2, and osteoprotegerin (OPG) via histone deacetylase (HDAC) regulation. Additionally, SCFAs inhibit osteoclast differentiation and activity by suppressing receptor activator of nuclear factor-kappa B ligand (RANKL) expression, thereby maintaining bone homeostasis. 4) Angiogenesis: SCFAs promote angiogenesis by upregulating vascular endothelial growth factor (VEGF), peroxisome proliferator-activated receptor gamma (PPARγ), and endothelial nitric oxide synthase (eNOS) expression. This is achieved through interactions with the mammalian target of rapamycin (mTOR) pathway, facilitating the formation of new blood vessels to supply oxygen and nutrients to the healing fracture site. 5) Inflammatory Response and Immune Cell Regulation: SCFAs modulate the inflammatory response by promoting regulatory T cell (Treg) differentiation, enhancing antimicrobial activity, and inducing an M2 macrophage phenotype. They regulate the production of pro-inflammatory cytokines such as tumor necrosis factor-alpha (TNF-α) and anti-inflammatory cytokines such as interleukin-10 (IL-10) and transforming growth factor-beta (TGF-β) through HDAC and histone H3 lysine 9 (H3K9) acetylation, thereby balancing the immune response and promoting tissue repair. 6) Chondrogenesis: SCFAs influence chondrogenesis by modulating the expression of pro-inflammatory cytokines, Sox9, Runx2, collagen type II alpha 1 (COL2A1), and small proteoglycans (sPGs) via GPR43 signaling. This regulation promotes the formation of cartilaginous tissue, which serves as a template for bone formation during fracture healing.

Significant observations have been made regarding the major impact of microbiota-derived metabolites on the process of bone repair (Fig. 2). The phenomenon of bone healing is a complex and highly regulated biological process that involves the restoration of structural and functional integrity in bones that have sustained injury or fracture [66]. Recent studies have unveiled that the gut microbiota and its associated metabolites can exert both direct and indirect influences on the process of bone repair. The effect of microbiota-derived metabolites on the host can substantially impact the fracture-healing process. The potential impact of microbiota metabolites on fracture healing can be linked to their indirect influence on the body's immune system [66]. Furthermore, microbiota-derived metabolites that are distributed systematically can reach considerable concentrations in bone marrow, directly impacting cells involved in the fracture healing process. The formation of hematomas and the process of fibrin clotting are critical components of the first phase of fracture healing. A study conducted by Kooistra et al. [147] demonstrated that butyrate can impact the breakdown of fibrin by promoting the creation of tissue-type plasminogen-activator (t-PA) in human endothelial cells *in vitro*. Imoto et al. [148] found that acetate, propionate, butyrate, and valerate stimulated t-PA production in primary human epithelial cells by activating GPR41 and GPR43. The presence of t-PA in the extrinsic fibrinolytic pathway is critical because it enhances the conversion of plasminogen to its active form, plasmin. Plasmin is in charge of fibrin breakdown. Plasmin, in addition to its involvement in fibrinolysis, is important in tissue repair processes such as bone fracture healing. This engagement includes a variety of modalities, including stem cell homing [149]. Valproate has been demonstrated to reduce the formation of hematoma and fibrin clotting in the context of intra-abdominal lesions. In the context of a research study, a single intraperitoneal dose of 50 mg/kg valproate was provided following the induction of peritoneal ischemia buttons in a rat model [66]. The results indicated a significant reduction of adhesions, as well as decreased levels of fibrinogen and vascular endothelial growth factor (VEGF) by approximately 50%, 56%, and 25%, respectively, in the collected button tissue when compared to the control group. This implies that metabolites produced by bacteria can potentially impact the bone repair process by altering the pathways responsible for fibrin breakdown and controlling the levels of VEGF.

Fibroblasts demonstrate migratory tendencies towards the location of a fracture, where they subsequently engage in the secretion and deposition of the initial constituents of collagen and proteoglycans, which are frequently detected in granulated tissue [66]. Numerous studies have shown that butyrate and propionate negatively impact the viability and development of gingival fibroblasts *in vitro* [[150], [151], [152], [153]]. In-vitro studies have demonstrated the ability of butyrate and propionate to trigger apoptosis and cytostasis in normal and inflamed gingival fibroblasts. These effects have been observed in original human cells as well as in cell lines. Studies with dosages ranging from 0.2 to 16 mM have documented the indicated effects. However, one study observed this effect only in inflammatory primary gingival fibroblasts, with no effect found in healthy primary gingival fibroblasts [151]. Maeshige et al. [154] demonstrated that the presence of butyrate at doses of 1 mM, 4 mM, and 16 mM resulted in the inhibition of pro-inflammatory IL-6 and pro-fibrotic factor expression in human dermal fibroblasts. On the other hand, a study by Karna et al. [155] showed that the application of 4 mM sodium butyrate, specifically the sodium salt variant of butyrate, promoted collagen biosynthesis in human skin fibroblasts *in vitro*. This increase in collagen formation coincided with an increase in the expression of the IGF-1 receptor. These findings suggest lower concentrations of butyrate and propionate may improve fibroblast survival, proliferation, and migration. The link between gut microbiota and bone healing is a complex and diversified phenomenon; nonetheless, new data suggest that metabolites generated by bacteria might considerably impact bone healing results.

### 3.7 Microbiota metabolites and osteoporosis

Microbiota-derived metabolites have been demonstrated to impact osteoporosis significantly, a metabolic bone condition characterized by decreased bone density and increased bone fragility, resulting in an increased risk of fractures [156,157]. Numerous studies have been carried out to examine the relationship between gut microbiota and the synthesis of various metabolites, providing insight into their functions in the development of osteoporosis [26].

The gut microbiome is critical in the breakdown of dietary components. Bacteria are known to be engaged in a variety of metabolic activities. As a result, dysbiosis of the gut microbiota impacts the metabolism of food components and causes changes in specific molecules produced by the host [158]. After establishing the presence of gut epithelial barrier defects in a rat model of osteoporosis produced by ethanol, Liu et al. [159] identified particular endogenous metabolites that exhibit differential expression. These metabolites could potentially serve as biomarkers for bone remodeling. The ethanol-treated group's serum and feces yielded 322 and 374 distinct metabolites, respectively [159]. The exclusion of differentially enriched metabolites detected in both ethanol and antibiotics can be attributed to the absence of phenotypic effects of antibiotics in osteoporosis, compared to the saline group. Finally, the analysis focuses on the top five and bottom five serum metabolites and fecal metabolites. According to KEGG analysis, the metabolites identified in this study are involved in various metabolic processes. Among the mechanisms involved are multiple amino acids, bile acid biosynthesis, purine and pyrimidine metabolism, alkaloids, and fatty acid metabolism [159]. The metabolic pathways enrichment study results indicate that tryptophan, serving as a central metabolite, is involved in several pathways. According to this observation, Liu et al. [159] also revealed heightened serum concentrations of serotonin and 2-(formylamino) benzoic acid, metabolites derived from tryptophan. Furthermore, Spearman correlation analysis revealed a statistically significant positive relationship between serum serotonin levels and the presence of six unique bacterial genera. When analyzing different metabolites in feces and serum, it was discovered that no similar molecules were discovered and extracted. This data challenges the concept that fecal metabolites can enter the bloodstream due to impaired gut epithelial barriers [160]. The observed outcome may be attributed to fluctuations in the metabolic rate of circulating metabolites and fecal wastes. The amount of scientific research available regarding the complex relationship between serotonin and bone remodeling is a topic that continues to generate ongoing discourse and controversy in the academic community [161]. According to research, the gut microbiota influences serotonin (5-HT) production by enterochromaffin cells and may have the ability to inhibit osteoblast proliferation [162]. Furthermore, it has been discovered that animals with colitis induced by DSS exhibit elevated levels of 5-HT, which are correlated with significant decreases in trabecular bone density [163].

Chevalier et al. [164] showed that a significant proportion of transcriptional alterations caused by ovariectomy in bone tissue are attenuated by exposure to elevated temperatures, with more than 90% of these changes being dampened. Additionally, the transplantation of warm-adapted microbiota into ovariectomized mice reduced approximately 59% of these transcriptional changes. Their findings indicated a significant protective impact of these interventions on the underlying bone changes associated with bone loss. They discovered that warmth affected the polyamine production pathway in the microbiota. This shows a possible link between these changes and their impact on bone mass and strength and other tissues altered by polyamine levels. The physiological relevance of increased polyamine synthesis mediated by microbiota in response to warmth suggests a broader impact beyond bone-related studies, potentially influencing various diseases associated with age and enhancing overall well-being [164]. They demonstrated that applying warmth exposure throughout later stages of development positively impacted bone microarchitecture and strength under normal physiological conditions. Chevalier et al. [164] also showed the potential applicability of this phenomenon in pathological conditions, demonstrating that it significantly mitigates the deleterious effects of estrogen deprivation in a murine model of osteoporosis. The observed improvements in bone structure are supported by changes in the gut bacteria composition caused by higher temperatures. These alterations help prevent bone loss, implying the presence of a microbiota-mediated communication link between warmth and bone health. Chevalier et al. [164] evaluated potential translation by undertaking human metadata analysis. The findings show a link between osteoporotic hip fractures and ambient temperature unaffected by vitamin D and calcium levels. Chevalier et al. [164] used a combination of combinatorial metagenomics, targeted metabolomics, and functional techniques to understand the mechanisms that increase the production of polyamines by thermoadapted microbiota, particularly acetylated spermidine, and putrescine.

A comparative analysis was performed by Stürznickel et al. [165] to identify the characteristics of patients who were diagnosed with primary sclerosing cholangitis (PSC) and demonstrated elevated bone mass or osteoporosis. An issue that emerged from these results concerned the possible influence of increased circulating bile acid levels on bone remodeling and the resulting effects on trabecular bone mass in patients with primary sclerosing cholangitis-associated osteoporosis. Prior investigations have suggested the existence of a possible correlation between bile acids and the functionality of diverse cell types that participate in the process of bone remodeling [166,167]. In addition, the farnesoid X receptor (FXR), which acts as an intracellular sensor for bile acids, affects bone production and resorption [105,168]. Thus, Stürznickel et al. [165] implemented a study to assess the impact of the two bile acids with the most significant elevated levels in the bloodstream of individuals diagnosed with PSCOPO. The researchers investigated the impact of unconjugated and conjugated forms of bile acids on the differentiation processes of osteoblasts and osteoclasts. Despite the little impact of UDCA, GUDCA, CDCA, or GCDCA on the osteogenic differentiation of hMSC-TERT cells, an interesting finding emerged concerning the transcriptional response of human osteoclasts to 50 μM GUDCA. This reaction displayed an unforeseen degree of specificity. The cell culture findings provided in this study may be considered significant since they demonstrate that the average blood concentration of GUDCA in the PSCOPO group was 25 μM, with two patients displaying levels beyond 50 μM [165]. The predominant effect of GUDCA was the inhibition of CTSK expression. Based on previous research findings, it is improbable that this result would play a role in the progression of PSCOPO disease, as previous studies have demonstrated that the inactivation or inhibition of CTSK results in an enhancement of bone mass [169]. In summary, the complex and diverse role of microbial metabolites in the development of osteoporosis involves the interaction of gut microbiota, metabolites, and bone health. Understanding these relationships can help develop innovative methods for the prevention and management of osteoporosis.

### 3.8 Microbiota metabolites and rheumatoid arthritis

Microbiota-derived metabolites appear to affect bone composition in experimental arthritic animals positively. Lucas et al. [98] employed three different experimental approaches in their study: direct supplementation with SCFAs, giving a high-fiber diet (HFD), and bacterial translocation. Their findings demonstrated that mice given direct SCFA supplementation and those fed an HFD had higher systemic bone density, lower bone resorption, and lower numbers of osteoclasts. The ability of SCFAs to inhibit the growth of osteoclasts and the process of bone resorption was revealed to be independent of the presence of the receptors gpr41 and gpr43. Nonetheless, both butyrate and propionate were shown to cause a metabolic shift toward osteoclast glycolysis while inhibiting TRAF6, a key signaling component implicated in osteoclastogenesis [170]. A further study revealed that SCFA treatment successfully decreased the severity of inflammation in arthritis. This medication also increased general bone density across the body and significantly decreased the expression of particular genes linked with osteoclasts in bone, such as TRAF6 and NFATc1 [98]. Butyrate has been shown to inhibit osteoclast differentiation and alleviate joint deformities associated with rheumatoid arthritis [171]. The potential mechanism might be connected with decreased histone deacetylase activity, which leads to changes in the KEGG pathways implicated in osteoclast formation [171]. SCFAs have a variety of immunological and metabolic activities via interacting with G protein-coupled receptors, most notably by activating the free fatty acid receptor 2 (FFAR2) [172]. Recent research has indicated that the manipulation of gut microbiota by administering prebiotics can enhance bone mass and mitigate skeletal disorders. There was a positive correlation between tibial strength and elevated propionate and butyrate concentrations in the cecum. According to numerous studies, a potential correlation also exists between the beneficial effects of prebiotics on bone mass and the production of SCFAs in the cecum. SCFAs can enhance phosphorus absorption, decrease gastric pH, and promote intestinal integrity. In turn, this inhibits the osteoclastic bone resorption process, which is facilitated by inflammatory cytokines, and decreases the expression of proinflammatory genes in the intestines and bone marrow [173]. The previously mentioned research emphasizes the importance of the gut-joint axis. Several studies so far have provided evidence of a link between rheumatoid arthritis and altered gut microbiota and SCFAs. Restoration of SCFA levels by regulating gut flora composition as an efficient tool to maintain intestinal barrier integrity, maintain immune homeostasis, reduce the inflammatory response associated with rheumatoid arthritis, and inhibit bone loss [174,175]. The findings presented in these studies demonstrate the crucial involvement of SCFAs in the interrelationship between the gastrointestinal tract and the joints.

Su et al. [176] employed untargeted metabolomics profiling to discern metabolic alterations in the feces and serum of individuals diagnosed with rheumatoid arthritis. Notably, their analysis revealed the presence of bile acids and tryptophan catabolites as significant metabolic modifications. A number of metabolites associated with rheumatoid arthritis, such as lipids, nucleotides, and amino acids, which were previously identified in studies by Guma et al. [177] and Tong et al. [178], were detected successfully; thus, the methodology of Su et al. [176] was validated. The secondary bile acids identified in the present study have not been subject to comprehensive studies in previous scientific literature. Su et al. [176] used a targeted metabolomics technique to investigate the bile acid content in both stool and serum samples from individuals with rheumatoid arthritis completely. Furthermore, secondary bile acids, such as UDCA, isoDCA, and isoLCA, have been found in rheumatoid arthritis patients, raising the idea that these compounds may influence the host's inflammatory response. Various studies have shown secondary bile acids to modulate peripheral regulatory T cells [[179], [180], [181]]. This phenomenon has been suggested to potentially play a role in the excessive activation of immune cells in the host. For example, it has been observed that UDCA effectively inhibits the development and activation of Treg cells, leading to a reduction in Treg-mediated immunosuppression [180]. Meanwhile, it has been discovered that isoDCA promotes the differentiation of Treg cells that have been triggered peripherally. Furthermore, Hang et al. [179] found that 3-oxoLCA and isoLCA could inhibit the growth of T helper 17 (TH17) cells. UDCA and a reduction in isoDCA, isoLCA, and 3-oxoLCA imply an imbalance in peripheral regulatory T-cell modulators in rheumatoid arthritis patients. Interestingly, these metabolite changes are consistent with key microbial biological aspects in the pathogenesis of rheumatoid arthritis. In summary, the integration of 16S sequencing, shotgun metagenomics, untargeted metabolomics, and targeted metabolomics provided a detailed understanding of the gut microbiome and microbial metabolome changes during the progression of rheumatoid arthritis. According to current knowledge, Su et al. [176] performed an initial study to document the involvement of bile acids derived from the microbiome in the pathogenesis of rheumatoid arthritis. A comprehensive examination of secondary bile acids, which have been comparatively overlooked in the realm of rheumatoid arthritis, was provided. As confirmed by additional cohorts, bile acids produced by the microbiome may potentially enhance the immune response and influence bone characteristics in individuals with rheumatoid arthritis despite the relatively small sample size of their study. For future research into the treatment of rheumatoid arthritis, the discovery has the potential to provide significant insights.

An association has been identified between the accumulation of kynurenine, a metabolite of tryptophan, and age-related bone loss [182]. A crucial determinant in the *in vitro* differentiation of MSCs into the osteoblast lineage is the kynurenine pathway activation [183]. The prevention and treatment of bone degradation may become a substantial objective in subsequent eras. Additionally, it is worth noting that a negligible percentage of tryptophan (approximately 1–2%) possesses the potential to produce serotonin in enterochromaffin cells via the mechanism mediated by tryptophan hydroxylase 1 (TPH1). SCFAs additionally regulate TPH1 expression. The significance of gut-derived serotonin in rheumatoid arthritis remains the subject of ongoing research.

On the contrary, there is speculation that serotonin could play a function in regulating bone remodeling [138,139], suggesting the existence of a gut-bone axis [184,185]. In conclusion, the metabolic processes involving tryptophan play a crucial role in the human body. Recent empirical research has provided insights into the complex relationship involving the microbiota, dietary patterns, genetic variables, mucosal integrity, and immune responses. However, the complex interplay between these factors presents a significant difficulty in determining a definitive hierarchy. Further research is required to understand better the underlying processes that govern the interactions between microbiota and hosts. This will contribute to refining target identification and treatment strategies, ultimately improving their accuracy and effectiveness.

### 3.9 Microbial metabolites and bone infection

Microbiota-derived metabolites are of significant importance in the context of osteomyelitis, a condition characterized by bone infections [186]. Osteomyelitis is a complex and frequently arduous medical disease characterized by the presence of bacterial infection within bone tissue. Microbiota-derived metabolites play a significant role in the etiology, diagnosis, and therapy of bone infections. Bui et al. [186] provided an overview of the association between microbial metabolites and bone infection in a study. The study presented empirical findings indicating that the administration of oligofructose led to a decrease in bacterial quantity in the infected tibia and soft tissue of mice exhibiting obesity and type 2 diabetes (T2D) compared to the control group. On the other hand, the administration of oligofructose did not produce any noteworthy impact on the dimensions of soft-tissue abscesses or the number of bacteria in lean/control + OF mice in comparison to lean/control + CL mice that were given cellulose as the control fiber. Based on the observation that lean/control mice, without any supplementation, did not exhibit gut dysbiosis or the associated inflammation commonly observed in cases of obesity and T2D, it can be inferred that the potential benefits of oligofructose may not substantially alter the course of infection, as lean/control animals already display characteristic inflammatory responses. In a study conducted by Bui et al. [186], it was discovered that oligofructose reduced the heightened levels of overall *Staphylococcus aureus* community colonization in mice with obesity and type 2 diabetes. Previous studies have shown that obesity and T2D have a significant impact on osteolysis in mice. Bui et al. [186] evaluated the influence of oligofructose in the setting of acute osteomyelitis, a condition characterized by transitory inflammation, in their study. At 21 days post-infection, the emphasis was on chronic infections, which have distinct characteristics such as fibrotic marrow and periosteal reactive bone growth [187]. However, it can be deduced that oligofructose had beneficial effects on infection outcomes in mice with obesity and T2D, suggesting the need for further investigation into its potential as a useful agent in regulating immunity and resolving gut dysbiosis. Bui et al. [186] analyzed the longitudinal changes in both the gut microbiota and metabolite profiles across all four groups of mice to determine a potential mechanism driving the reduction in inflammation and severity of infection. In the absence of supplementation, the gut microbiota of obese/T2D mice revealed a dysbiotic profile identifiable from that of lean/control animals. Significant changes in the gut microbiota were found in both lean/control and obese/T2D mice after oligofructose treatment, whereas cellulose, a control fiber, had no effect. In summary, Bui et al. [186] successfully demonstrated the therapeutic effectiveness of oligofructose, a form of dietary fiber, in treating osteomyelitis in obese and T2D hosts. In obese and T2D mice, oligofructose treatment lowered bacterial load, reduced *S. aureus* colonization of the bone, and moderated the hyper-inflammatory response. These data show that dietary fiber may have reduced the disease severity. The recorded findings revealed a link between changes in the gut microbiota and general metabolic processes, indicating the potential role of polyamines in boosting infection response. The increased predominance of *Bifidobacterium pseudolongum* and other oligofructose-responsive bacteria is expected to contribute to increased polyamine synthesis, given their abundance is linked to greater levels of acetyl-ornithine [186]. Acetyl-ornithine serves as an intermediate component in the biosynthesis of polyamines, a class of organic compounds. Notably, this substance is solely synthesized by bacterial organisms. The administration of polyamines, namely spermine and spermidine, by oral ingestion has demonstrated compelling data on their potential to reduce the severity of infection in patients diagnosed with obesity and T2D. In conclusion, our discovery has unveiled a hitherto unidentified role of oligofructose and polyamines within the realm of bone infections. This discovery emphasizes the necessity for further investigation into their role in immune regulation and their potential as adjunctive therapies for persons with obesity and T2D who are at risk for invasive *S. aureus*-induced osteomyelitis.

Inflammatory conditions linked to bacterial infection, such as periodontitis, osteomyelitis, and some types of arthritis, are characterized by pronounced bone loss due to increased bone resorption [188]. The osteoclast development process entails several sequential events, including cellular interactions, fusion, and subsequent differentiation. RANKL and macrophage-colony stimulating factor (M-CSF) are commonly acknowledged as significant and effective factors in the osteoclastogenesis process [[189], [190], [191]]. Within macrophages, lipopolysaccharide (LPS) promoted the synthesis of many cytokines and mediators, including TNF-α, IL-1β, and prostaglandin E2 (PGE2). These compounds have been shown to play an important role in developing osteoclast progenitors and subsequent bone resorption [192,193]. Therefore, it appears that LPS has a complex impact on the process of osteoclastogenesis. Islam et al. [194] provided evidence that the administration of LPS induces TRAP-positive multinucleated giant cells (MGC) development in RAW 264.7 cells. These MGCs also displayed the ability to form pits on calcium carbonate-coated plates. As a result, it is strongly advised to use LPS to increase the production of osteoclasts in RAW 264.7 cells [194]. The use of the RAW 264.7 cell line has been applied in scientific investigations to explore the mechanism underlying osteoclastogenesis induced by RANKL [195,196]. Islam et al. [194] provided evidence to support the idea that RAW 264.7 cells can behave as osteoclast progenitors and differentiate into osteoclasts when exposed to 10.13039/501100012274LPS. LPS has been shown to increase the viability and merging of osteoclast precursor cells in the absence of RANKL. Furthermore, when M-CSF is present, LPS has been shown to enhance pit development. Furthermore, LPS was found to trigger osteoclast formation in mouse osteoblasts and bone marrow cell co-cultures [194]. LPS, when combined with dexamethasone, increases osteoclast formation in cultures of whole bone marrow cells. Furthermore, combining dexamethasone with 1,25-dihydroxyvitamin D3 greatly increases this impact [197]. In contrast, it has been observed that LPS effectively suppresses the development of osteoclasts in whole bone marrow cells when combined with 1,25-dihydroxyvitamin D3, mostly through the synthesis of GM-CSF [197]. Therefore, it may be inferred that LPS exerts a multifaceted influence on the process of osteoclastogenesis. Their findings show that LPS may independently stimulate osteoclastogenesis in RAW 264.7 cells. LPS-induced osteoclastogenesis may be substantially connected to LPS's bone resorption activity. The results showed that LPS induces the growth of osteoclasts in RAW 264.7 cells but not in mouse peritoneal cells. As a result, RAW 264.7 cells may display monocytic progenitor cell features. Alternatively, a lack of specific chemicals may result in poor maturation of peritoneal cells. The stimulation of osteoclast development by LPS has the potential to be a useful experimental paradigm for studying and comprehending the process of osteoclastogenesis. Finally, our data highlight the importance of microbial metabolites in developing and treating bone infections, especially osteomyelitis. The findings suggest that interventions aiming at altering the gut microbiota, such as dietary fiber supplementation with oligofructose, can potentially improve infection outcomes. Furthermore, the effect of microbial elements such as LPS on bone resorption and osteoclast development reveals the complicated relationship between microorganisms and bone health maintenance.

### 3.10 Microbiota metabolites and metastatic cancer to bone

The study of microbiota metabolites and metastatic cancer in bone is a growing field that sheds light on the complex relationship between gut microbiota, the host's immune system, and cancer [198]. The occurrence of metastatic cancer in the bone, which often comes from primary tumors elsewhere in the body, poses a significant treatment challenge. The skeletal system is commonly used as a metastatic site for solid tumors [198]. The particular mechanisms by which gut microbiota impacts the dissemination of solid tumors to extra-intestinal sites, particularly the bones, have not been well explored in scientific research. Nevertheless, increasing empirical data indicates that the gut microbiota exerts influence over the bone microenvironment and contributes to preserving skeletal health. The function of the gut microbiota in affecting bone diseases, including osteoarthritis and osteoporosis, has been demonstrated through the brain-gut-bone axis [4,19]. The brain-gut-bone axis, alternatively referred to as the gut-bone axis, includes the intricate network of communication between the brain/nervous system, the gut bacteria, and the skeletal system, culminating in a discernible influence on bone health [199,200]. The presence of increased osteoclast activity in people with osteoarthritis and osteoporosis is thought to play an essential role in the development of bone metastasis by promoting tumor cell penetration into the bone microenvironment. The study found that mice with arthritis had a higher incidence of bone metastases caused by breast cancer cells than mice without arthritis [201]. Furthermore, while osteoporosis was not recognized as a risk factor for bone metastasis, research has shown that untreated osteoporosis in breast cancer patients might hasten the onset of bone metastasis [202].

In cases of dysbiosis, LPS from the gut microbiota is transferred into the circulation. LPS in the circulation causes monocytes and macrophages to differentiate into osteoclasts while promoting the development and lifespan of these osteoclasts [203,204]. Previous studies have reported that increased levels of LPS in the bloodstream can induce the release of the proinflammatory cytokine TNF-α from macrophages. The cytokine in question is acknowledged for its capacity to modulate the actions of osteoclasts by means of its interaction with TNFR-1 [203,205,206]. LPS-induced TNF-α has been shown to accelerate bone resorption by boosting the generation of osteoclasts in a murine macrophage cell line known as RAW 264.7 cells without activating the RANK/RANKL pathway [206]. A potential mechanism for LPS-induced osteoclastogenesis is the upregulation of the nuclear factor of activated T cell transcription factor c1 (NFATc1). This transcription factor is essential for osteoclast cell growth and is thought to be found in the nucleus of osteoclast progenitors [207,208]. In addition to these concepts, it has been discovered that LPS treatments reduce bone density in two chronically inflammatory rat models, as well as in a rodent model of mice lacking the prostaglandin E receptor 4 (EP4), which is responsible for regulating osteoclast formation on osteoblasts [209,210]. Furthermore, it has been demonstrated that the LPS-Toll-like receptor 4 (TLR4) axis contributes to the severity of osteoporosis and osteoarthritis [211]. Aside from its involvement in the circulation of LPS, the gut microbiota can also control osteoclastogenesis through bile acid metabolism. Lithocholic acid (LCA) primarily promotes the process of osteoclastogenesis or the production of osteoclasts. LCA is a secondary bile acid formed by the 7-alpha-dehydroxylation pathway from chenodeoxycholic acid. Bacterial hydrolases found on the cell walls of gut bacteria have a substantial influence on this conversion process [212,213]. LCA becomes a ligand for the vitamin D receptor after being converted. Historically, the vitamin D receptor, which is responsible for binding with vitamin D, has been linked to calcium absorption. Furthermore, there is a link between increased vitamin D levels and increased bone mineral density [214,215]. Nevertheless, the binding of LCA to the vitamin D receptor on osteoblasts leads to the inhibition of bone growth [216]. LCA has been observed to affect osteoblasts directly, leading to the loss of bone tissue [167,217]. The results of these studies suggest that the process of osteoclastogenesis is influenced to some extent by LPS and LCA generated by the gut microbiota, which has systemic effects. In summary, investigating the relationship between metabolites generated by microbiota and the development of metastatic cancer in the skeletal system is an intriguing and evolving field of research. The examination of the influence of microbial metabolites on the bone microenvironment, immune responses, and cancer progression is a potential avenue for discovering novel treatment approaches for individuals afflicted with bone metastases. Further research must elucidate the specific metabolites and processes involved in the complex interplay between microbiome and bone metastasis cancer.

## 4 Microbiota derived metabolite-based therapy for bone disorders

The use of microbial metabolites as a therapeutic method for bone disorders is a growing field of study that has the potential to cure a variety of skeletal-related disorders. Recent research has shown that two unique microbial metabolites, sodium butyrate and ursodeoxycholic acid (UDCA), offer promising therapeutic capacities in the context of bone diseases [218]. UDCA regulates intracellular reactive oxygen species (ROS) and pro-inflammatory cytokines [[219], [220], [221]]. The presence of intracellular ROS promotes the activation of mitochondrial permeability transition pores (MPTPs), which are important in triggering cellular death [221]. UDCA has decreased ROS generation, MPTP formation, and the expression of pro-apoptotic proteins, including the transcriptional regulator of p53 [221]. In addition, UDCA, a compound recognized for its anti-inflammatory properties, facilitates bone regeneration by alleviating the detrimental effects linked to inflammation at the site of the lesion [222,223]. PUDCA NPs, which are polymeric nanoparticles (NPs) that are sensitive to hydrogen peroxide (H2O2), enable the elimination of H2O2 from defect lesions upon application. Furthermore, even in the presence of H2O2, persistent UDCA release from PUDCA NPs is possible. UDCA has been found to efficiently reduce the intracellular amount of ROS in cells, hence generating an environment suitable for improved bone regeneration. Furthermore, H2O2 near the location of the defective lesion, as well as consecutive cell culture in a two-dimensional environment, might trigger intracellular ROS [224]. Under normal growth conditions, nanoparticles of UDCA and 3,7-dihydroxy-12-oxo-5-cholan-24-oic acid (PUDCA) were shown to reduce the intracellular level of ROS in MSCs. PUDCA NPs can promote MSC osteogenic differentiation even in the absence of H2O2 by reducing the high intracellular ROS that is already present owing to sequential cell growth. In brief, Arai et al. [218] developed nanoparticles encapsulating bone-regenerating PUDCA that can eliminate excess H2O2 and gradually release UDCA when exposed to H2O2. The use of collagen sponges with PUDCA NPs increased bone healing in both the epiphyseal and diaphysis regions of long bones in mice while simultaneously reducing the inflammatory response. The efficacy of UDCA and PUDCA NPs to induce bone regeneration is considerably enhanced by the scavenging of H2O2. As a result, it is reasonable to regard UDCA and PUDCA NPs as very effective pharmacological agents for bone regeneration.

Additionally, based on the findings, it is evident that sodium butyrate plays a significant role in therapy for bone disorders through multiple mechanisms. Directly, sodium butyrate promotes bone formation and inhibits bone resorption. Its ability to stimulate bone formation in the gut–bone axis and its pivotal role in parathyroid hormone-dependent bone formation highlight its importance in maintaining bone health [225]. Indirectly, sodium butyrate interacts with immune cells, particularly Tregs, locally in the gut and systemically, leading to immunomodulatory effects [225]. These effects involve the production of immunosuppressive cytokines and interactions with bone marrow CD8+T cells, ultimately promoting bone anabolism and suppressing bone resorption. Butyrate enhances the suppressive function of Treg cells, as demonstrated by their ability to increase NFAT and SMAD binding to the Wnt10b promoter and subsequent Wnt10b production in CD8^+^ T cells in coculture experiments [80]. In conclusion, sodium butyrate exerts its therapeutic effects on bone disorders by promoting Treg cell differentiation, enhancing Wnt10b production, and modulating signaling pathways involved in bone metabolism.

The etiology of rheumatoid arthritis involves epigenetic modification of cellular proteins, particularly through acetylation [92]. The aforementioned alteration plays a crucial function in the regulation of signaling pathways and the transcription of key factors, including T-bet, Gata 3, retinoic-acid-receptor-related orphan nuclear receptor gamma (RORγt), and forkhead box P3 (FOXP3). The previously mentioned variables substantially influence the formation and functionality of effector T cells. In order to provide additional insight, it is conceivable that the modulation of gene expression or protein modification in effector T cells may be associated with autoimmune diseases. In a manner similar to butyrate, the pan-HDAC inhibitors SAHA and TSA have exhibited therapeutic efficacy in the context of rheumatoid arthritis [92]. Nevertheless, the precise targets and methods by which these inhibitors exert their therapeutic benefits have yet to be determined. The introduction of butyrate to mice with collagen-induced arthritis (CIA) led to the recovery of many indicators linked to joint impairment, inflammation, the generation of proinflammatory cytokines, and the development of osteoclasts. The mechanism behind the impact of butyrate on osteoclastogenesis and effector T-cell differentiation was elucidated by Kim et al. [92]. The mechanisms above are of paramount importance in the pathogenesis of rheumatoid arthritis. The depletion of HDAC7 has been observed to decrease bone degradation, whilst the lack of HDAC3 has been linked to an elevation in bone resorption [96,226]. The inhibition of osteoclastogenesis by HDAC9 occurs through a distinct mechanism compared to HDAC3 [227]. It was found to be involved in the expression of HDAC2 during the process of osteoclastogenesis. The process of osteoclastogenesis, initiated by RANKL, is enhanced by HDAC2 overexpression through Akt activation. Activation of a specific process leads to the downregulation of FoxO1, while blocking HDAC2 inhibits the development of TRAP-positive osteoclasts *in vitro* [228]. The downregulation of TRAP expression was noted in response to butyrate treatment, indicating a possible mechanism by which the HDAC2-associated GR-SLPI axis modulates osteoclastogenesis. The Akt protein negatively regulates the expression of GR [229]. As a result, suppressing the HDAC2-related pathway can potentially disrupt the Akt-related pathway, resulting in osteoclast differentiation. Butyrate inhibited the expression of HDAC2, resulting in the suppression of GR transcription via deacetylation. As a result, SLPI expression was increased. The SLPI protein protects epithelial tissue from serine proteases and has been shown to have anti-inflammatory properties and to reduce joint damage in cases of arthritis [[230], [231], [232]]. It was demonstrated that the upregulation of SLPI expression within osteoclasts occurs when HDAC2 is inhibited using butyrate. Butyrate injection reduced the amount of TH17 cells while increasing the number of T reg cells, as reported by Kim et al. [92]. This therapeutic effect was reported in a mouse arthritis model and was linked to *in vivo* and *in vitro* modification of the TH17/Treg balance. Butyrate regulation of the TH17/Treg balance was discovered to be independent of the Signal transducer and activator of transcription 3 (STAT3) phosphorylation, a known regulator of the TH17/Treg balance. Butyrate's manipulation of the TH17/Treg balance may have influenced the expression of IL-10, resulting in reduced therapeutic effectiveness of butyrate in mice missing IL-10. Furthermore, butyrate injection resulted in T-cell population modification within human peripheral blood mononuclear cells (PBMCs) as well as inhibitory effects on osteoclastogenesis [92]. Butyrate appears to offer promise as a prospective therapeutic method for the treatment of rheumatoid arthritis, according to these data. These findings suggest that sodium butyrate, a component of the gut microbiota, reduces rheumatoid inflammation by targeting HDAC2 in osteoclasts and HDAC8 in T cells. As a result, sodium butyrate has the potential to be used in the treatment of rheumatoid arthritis. In conclusion, studying microbial metabolites such as sodium butyrate and UDCA as possible treatments for bone diseases provides a compelling and promising approach to developing innovative therapeutic techniques. The effectiveness of these metabolites in regulating immune responses, reducing inflammation, and promoting bone regeneration suggests that they have the potential to be significant therapeutic choices for the treatment of diseases such as rheumatoid arthritis and bone abnormalities. Nonetheless, further research and thorough clinical trials are required to fully understand and harness their therapeutic potential in the context of bone-related disorders.

## 5 Concluding remarks and future directions

The intricate and dynamic domain of microbiota-derived metabolites in relation to bone health has unveiled their substantial impact on both healthy bones and diseases. Microbiota-derived metabolites, namely SCFAs, play a crucial role in facilitating mineral uptake, promoting bone mineralization, and facilitating the production of vitamin K. In addition, recent studies have unveiled the significant impact of microbial SCFAs like butyrate and propionate, on various aspects of mineral absorption, osteogenic differentiation, chondrogenesis, bone formation, and resorption. Specifically, SCFAs have been implicated in microbiota-induced changes in IGF-1 levels and bone remodeling. Studies have demonstrated that antibiotic-treated mice supplemented with SCFAs showed similar effects on IGF-1 levels and bone mass as colonized mice. Further research on GF mice administered SCFAs or lacking SCFA receptors may shed more light on the effects of SCFAs on bone health. These metabolites influence crucial cellular processes such as osteoclast differentiation and metabolic alterations in osteoclast precursors, ultimately shaping skeletal homeostasis.

Studies have shown a positive association between blood concentrations of bile acids and BMD in postmenopausal women, indicating the potential impact of bile acids on bone health. The gut microbiota can modulate the quantity and composition of these bile acids, influencing bone metabolism through receptors like the FXR and TGR5. Cellular processes crucial for bone health, including osteoclast differentiation, are significantly influenced by cellular metabolism. Research has shown that stimulating certain microbial metabolites like propionate and butyrate can lead to metabolic alterations in osteoclast precursor cells, particularly a shift toward glycolysis. This metabolic change induces cellular stress, ultimately impeding the process of osteoclast differentiation.

Furthermore, SCFAs and other microbial metabolites have been found to reduce the severity of conditions such as rheumatoid arthritis and osteoporosis. They also influence bone remodeling and serotonin levels derived from the intestines. Notwithstanding their relatively low levels of circulation, these substances exert significant indirect impacts, influencing immune cells and inflammatory responses. The gastrointestinal microbiota is known to impact bone health significantly and may even contribute to the progression of bone metastasis. Secondary bile acids and LPS are involved in these processes. LPS, produced by the intestine's microbiota, enters the bloodstream and induces osteoclast differentiation and inflammation. As an alternative, LCA damages osteoblasts and inhibits bone formation; it is a secondary bile acid. Investigating microbial metabolites as a potential therapeutic approach to treat rheumatoid arthritis and bone disorders is auspicious. Particular metabolites, including sodium butyrate and UDCA, have demonstrated promise in the context of rheumatoid inflammation reduction and bone regeneration. They facilitate osteogenic differentiation, reduce inflammation, and reinstate immune system balance through various mechanisms. Nevertheless, additional investigation and clinical testing are imperative to exploit microbial metabolites' therapeutic capabilities fully.

Moving forward, further investigation into the mechanisms underlying the effects of microbial metabolites on bone is warranted. Clarifying the specific signaling pathways and molecular interactions involved in these processes could unveil novel therapeutic targets for preventing and treating bone disorders such as osteoporosis and osteoarthritis. Additionally, exploring the potential synergistic effects of microbial metabolites with conventional treatments or dietary interventions could lead to more effective strategies for promoting musculoskeletal health. In essence, the study of microbial metabolites in the context of bone metabolism represents a burgeoning field with immense potential for advancing our understanding of musculoskeletal health and disease. Continued exploration and innovation in this area are essential for unlocking new therapeutic strategies and improving outcomes for individuals with bone disorders.

## Additional information

No additional information is available for this paper.

## Data availability statement

No data was used for the research described in the article.

## Funding

None.

## Ethics statement

Review and/or approval by an ethics committee was not needed for this study because [This work is a review of the literature and does not address the ethical considerations of animal, cell, and human experimentation.].

## CRediT authorship contribution statement

**Dong Han:** Writing – original draft. **Weijiao Wang:** Writing – original draft, Visualization. **Jinpeng Gong:** Writing – review & editing, Supervision. **Yupeng Ma:** Writing – review & editing. **Yu Li:** Writing – review & editing.

## Declaration of competing interest

None.
